# Factors affecting the seasonal distribution and biomass of *E*. *pacifica* and *T*. *spinifera* along the Pacific coast of Canada: A spatiotemporal modelling approach

**DOI:** 10.1371/journal.pone.0249818

**Published:** 2021-05-14

**Authors:** Rhian Evans, Philina A. English, Sean C. Anderson, Stéphane Gauthier, Clifford L. K. Robinson

**Affiliations:** 1 Fisheries and Oceans Canada, Institute of Ocean Sciences, Sidney, British Columbia, Canada; 2 Fisheries and Oceans Canada, Pacific Biological Station, Nanaimo, British Columbia, Canada; 3 Department of Mathematics, Simon Fraser University, Burnaby, British Columbia, Canada; Swedish University of Agricultural Sciences and Swedish Institute for the Marine Environment, University of Gothenburg, SWEDEN

## Abstract

Euphausiids are a keystone species in coastal food webs due to their high lipid content and seasonally high biomass. Understanding the habitat and environmental drivers that lead to areas of high biomass, or ‘hotspots’, and their seasonal persistence, will support the identification of important foraging regions for mid- and upper- trophic level predators. We quantify the distribution of hotspots of the two dominant species of euphausiid in the north-east Pacific Ocean: *Euphausia pacifica* and *Thysanoessa spinifera*, as well as euphausiid larvae (mixed species). The Canadian coast encompasses the northern California Current Ecosystem and the transition zone to the Alaska current, and is a highly productive region for fisheries, marine mammals, and seabirds. We used spatiotemporal modelling to predict the distribution of these three euphausiid groups in relation to geomorphic and environmental variables during the important spring-summer months (April through September) when euphausiid biomass is highest. We quantified the area, intensity, and persistence of biomass hotspots across months according to specific oceanographic ecosections developed for marine spatial planning purposes. Persistent hotspots of both adult species were predicted to occur along the 200 m depth contour of the continental slope; however, differences were predicted on the shallower Dixon shelf, which was a key area for *T*. *spinifera*, and within the Juan de Fuca Eddy system where *E*. *pacifica* hotspots occurred. The continental slope along the west coast of Vancouver Island was the only persistent hotspot region common between both adult species and euphausiid larvae. Larval distribution was more correlated with *T*. *spinifera* than *E*. *pacifica* biomass. Hotspots of adults were more persistent across months than hotspots of euphausiid larvae, which were seasonally patchy. The persistence of biomass hotspots of forage species through periods of low overall biomass could maintain trophic connectivity through perturbation events and increase ecosystem resilience to climate change.

## Introduction

*Euphausia pacifica* and *Thysanoessa spinifera* are the most abundant euphausiid species in the north east Pacific Ocean [1–3]. Both species comprise a large proportion of the diet of numerous forage and commercially important fish species in this area, such as sardine (*Sardinops sagax*), anchovy (*Engraulis mordax*), Pacific herring (*Clupea pallasii*), Pacific hake (*Merluccius productus*) and salmon (Oncorhynchus spp.) [4], and a large proportion of seabird [1, 5] and whale diets [6, 7]. Euphausiids are an energy rich food source due to a high lipid content [8], and are seasonally abundant forming large aggregations or ‘swarms’. Zooplankton in general are considered indicator species of their environment as they show high sensitivity to changes in physical parameters due to short life spans. Large variability in the spatial and temporal distribution of *E*. *pacifica* and *T*. *spinifera* has been linked to seasonal changes in the timing and intensity of the spring transition, which is reflected by ocean temperature and productivity cycles [2, 9–11].

Euphausiid research in the California Current ecosystem (CCE) has focused on the physical mechanisms that lead to regions of elevated biomass, or ‘hotspots’, of euphausiids e.g. [12–18]. The distribution, intensity and temporal persistence of these areas has been quantitatively linked with local euphausiid predators [17, 19–24]. Physical processes that lead to hotspot formation tend to be localised and are linked to the timing and local intensity of upwelling and the complexity of seafloor topography [16]. Bathymetrical edges, such as along continental margins and around the edges of canyon systems, are regions where hotspots of euphausiids often occur [7, 15, 16, 25] and are therefore also often important foraging regions for their predators [6, 26]. Both *E*. *pacifica* and *T*. *spinifera* have been recorded to occur in order-of-magnitude larger densities along steep canyon edges than in surrounding water [7, 27]. *E*. *pacifica* is approximately 100 times more abundant than *T*. *spinifera* [10, 28], and they exhibit differences in life history, reproductive timing, organism size and habitat associations [e.g. 3, 10, 12, 29], which control their distributions.

The Canadian Pacific coast encompasses the transition zone between the California Upwelling Domain and the northerly Alaska Downwelling Domain, although a short upwelling season still occurs along the central and northern British Columbia (BC) coast [30]. The Juan de Fuca canyon off south west Vancouver Island is part of the upwelling domain and is an important region for euphausiids [25, 31, 32]. Whether hotspots of euphausiids are spatially and temporally persistent is unknown. The distribution of adults and larvae is spatially patchy and spatial segregation by age classes occurs perpendicular to the coast [3, 31, 32], with less-motile larvae more vulnerable to changes in current patterns [3]. As in the California Current ecosystem, *E*. *pacifica* is thought to be more numerically dominant overall, with *T*. *spinifera* more abundant over shallower areas on the shelf, and *E*. *pacifica* more abundant in deeper waters over the continental shelf and slope [12, 29, 33].

Monitoring of euphausiid dynamics for the Canadian coast has been spatially and temporally patchy, with a focus on the west coast of Vancouver Island [3, 34, 35]. Species-specific studies of the spatial structure of euphausiid hotspots in the northeast Pacific Ocean are few [but see 14]. It is currently unknown whether structural differences occur between aggregations of the two species, whether the distribution of these hotspots varies between species, and how temporally persistent the hotspots are after the spring transition when biomass is highest. The spatial structure and temporal patchiness of euphausiid larvae hotspots is even less well studied. Therefore, the main aim of this work was to identify areas of persistently high biomass of the two dominant species of euphausiids, and euphausiid larvae, using a long-term euphausiid net-capture dataset for the whole BC coast. Spatiotemporal modelling using geostatistical methods has been shown to generate more precise and accurate biomass and abundance predictions than design-based methods, or other modelling approaches used for species distribution modelling [36, 37]. Through these models, we identify 1) the physical habitat characteristics shaping euphausiid distribution; 2) where biologically important areas occur for the two species and euphausiid larvae; and 3) whether these hotspot areas are persistent through the spring and summer months. Given the known effects of complex bathymetry for balancing advective and retentive currents [16], we hypothesize that euphausiid hotspots will occur along depth isobars in areas such as the Juan de Fuca Eddy and canyon system and the continental slope. We compare the distribution of predicted hotspots across oceanographic ecosections for the British Columbia (BC) coast, which were developed for marine spatial planning purposes [38].

## Materials and methods

### Euphausiid biomass data

The Canadian government has collected net-based zooplankton samples to estimate abundance and biomass for the British Columbian coast for over 30 years [~1986-present, 32], as part of a variety of sampling programs. Prior to 1993 the spatial and temporal resolution of sampling was low; therefore, we truncated these years from the dataset prior to analysis. Data were collected by both vertical hauls and oblique hauls, which were considered comparable, however this will add some bias to results. We analyzed data from daylight hauls, as these were more numerous than night hauls. A correction factor [33], was applied to daylight counts to account for net avoidance by euphausiids.

**Fig 1:**
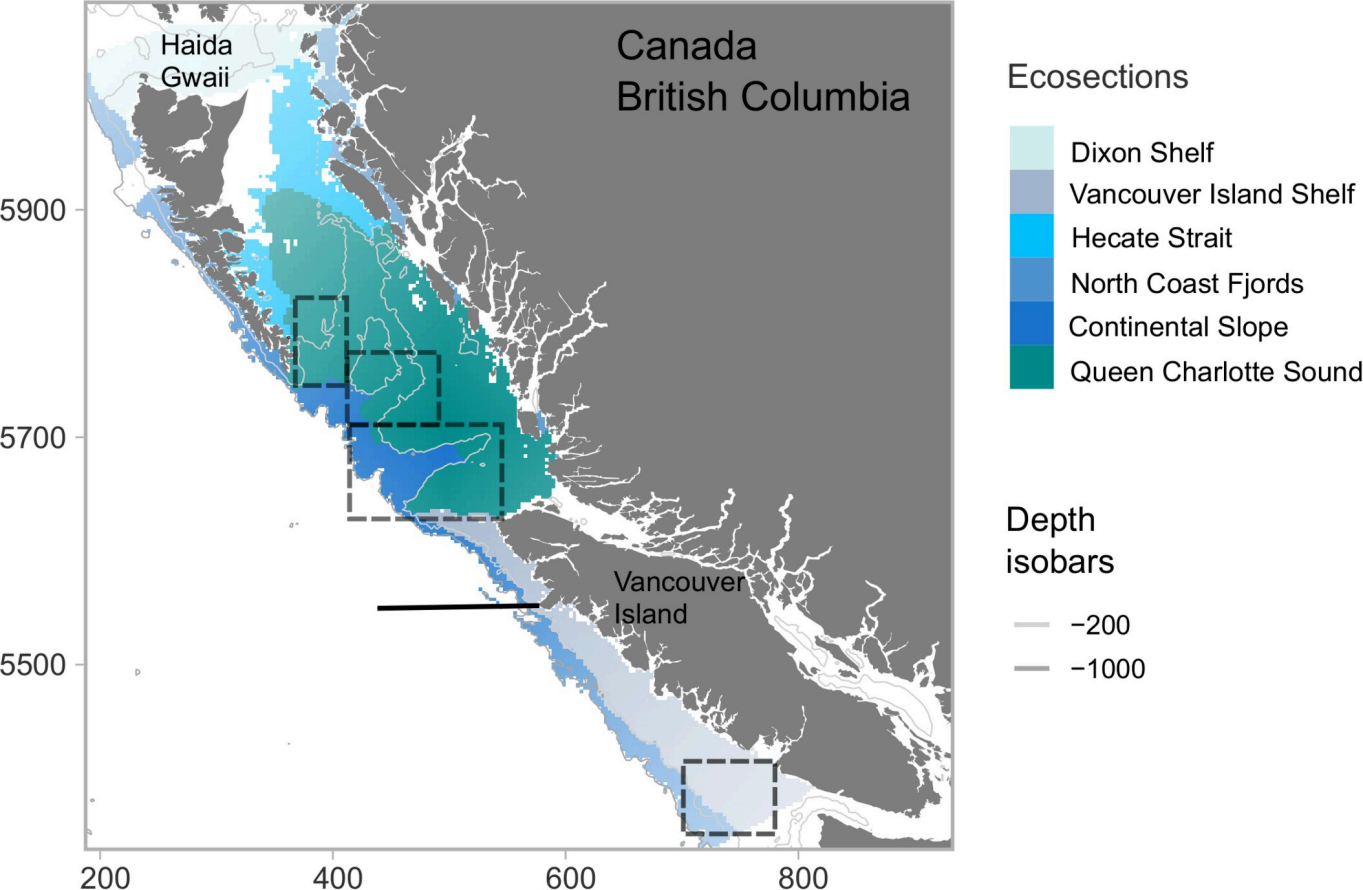
The area of the British Columbian shelf and slope considered in this study. Coloured sections show the boundaries of the BCMEC oceanographic ecosections. Grey lines indicate the 200 and 1000 m isobars. The solid black line shows the division of north and south bioregion sections [38]. The dashed black lines indicate regions of more complex bathymetry including the Juan de Fuca canyon and Eddy system on the Vancouver Island shelf and three sea valley and canyons systems on the west edge of Queen Charlotte Sound.

In general, nets were lowered to near the bottom (~+/-5m) or to a maximum of 250 meters. Within the dataset there were some points with large disparity between bottom depth and sampling depth, and these were excluded from further analysis: at a bottom depth of less than 100m, sampling points were excluded if they were over 30m from the bottom, while at bottom depths between 100m and 250m, sampling points were excluded if they were over 50m from the bottom. We assigned sampling sites to an oceanographic ecosection (Fig 1), based on similar biological and physical characteristics, developed as part of the British Columbia Marine Ecological Classification System (BCMEC) [39]. Due to known differences in currents along the slope, the continental slope ecosection was further divided into north and south bioregions using boundaries used by the Canadian government for marine spatial planning purposes [38].

For all stations sampled, formalin-preserved zooplankton samples were sub-sampled to contain ~300 individuals and identified to species level using a dissecting microscope. Euphausiids were separated from the rest of the plankton sample and measured. Larval stages were assigned a “stage” group comprised of eggs: euphausiid eggs and nauplii <3mm, s1: euphausiid zoea <5mm, s2: euphausiid juveniles ≥5mm<10mm, and S4: euphausiid ≥10mm, which were considered adults and separated into males and females. For volume calculations, a flow meter was used when available (+90% of samples), and sample volumes were compared with the expected value calculated from the amount of wire put out during sampling as a quality assurance check. Multiplicative species and stage-specific size coefficients (estimated mg dry weight per individual) were used to convert euphausiid abundance counts to biomass estimates [see Ref. 40 for coefficients]. Biomass values for each stage and sex were standardized via the volume to mg/m^3^. We later converted the euphausiid biomass from mg/m^3^ to vertically integrated dry weight biomass mg/m^2^ based on sampling depth, which is considered more robust to variability in sampling depth than cubic biomass estimates [41].

The majority of voyages occurred over the spring and summer months between April to September. This coincides with the important transition period for the upwelling domain of the coast [42] and the peak euphausiid biomass period [12, 29]. We focused on these months for further analysis. A map showing the distribution of sampling points across months (n = 1609) as modelled can be viewed in the (S2 Fig). The contribution of adult *E*. *pacifica* and *T*. *spinifera* to overall biomass was calculated as a ratio of adult euphausiid biomass (*E*. *pacifica*:*T*. *spinifera*).

### Spatiotemporal modelling of seasonal changes in biomass

We estimated *E*. *pacifica* and *T*. *spinifera* biomass using spatiotemporal models to explore differences in the environment and habitat preference of these two dominant euphausiid species. Larval stages of euphausiids are difficult to differentiate; therefore, a large proportion of larval stages in the database were not identified to the species level. Because of this, we were unable to separately model the distribution of species-specific larval stages; instead, we modelled the biomass of all larval stages combined. For all models, we chose biomass over abundance, as it is more ecologically relevant in terms of predator consumption.

The contribution of many sampling programs meant that the euphausiid biomass dataset was unbalanced and irregular both spatially and across seasons. We opted to account for this irregular sampling design using a model-based approach rather than a design-based approach, by fitting a geostatistical model that makes use of a predictive-process stochastic partial differential equation (SPDE) mesh. This ‘mesh’ approximates a Gaussian Markov random field (GMRF) through a set of representative locations (‘knots’) and bilinear interpolation between those knots [43]. We fit the models via the R package ‘sdmTMB’ [44, 45], which interfaces the integrated nested Laplace approximation [INLA; 46] with Template Model Builder [TMB; 47] to find the maximum marginal likelihood while integrating over spatiotemporal random effects. This method is increasingly popular in ecology [37, 48–50]. Using this approach, biomass is modelled as a function of both ‘fixed’ effects, as a result of explicit habitat variables such as depth, and of ‘random’ effects as a product of unobserved or ‘latent’ spatiotemporal effects using Gaussian Markov random fields. There is evidence that such model-based standardization of survey biomass index trends better encapsulates *in situ* spatial correlation and processes than design-based estimates [30, 41]. A thorough outline of the model framework and underlying statistical structure is provided [44]. We made some modifications to this framework, which are discussed in more detail below.

Our models are GAMMs (generalized additive mixed-effect models) with spatiotemporal Gaussian Markov random fields. The random fields account for spatial and temporal autocorrelation between sampling events, as well as estimating unmeasured components of habitat suitability, allowing that suitability to change through time [36]. The models estimate a spatiotemporal random field that controls for remaining correlated spatial correlation processes each month that are not accounted for by the fixed effects. This random field follows a stationary autoregressive (AR1) process with a first-order correlation (see Supporting information). To allow for a smooth relationship between some predictors and the response variable, we used thin plate regression splines [51] with fixed basis dimensions as calculated via the ‘mgcv’ R package [44, 45]. In all cases, sampling year was included as a factor (estimating a separate mean per year), and numeric month as a spline, to account for seasonal fluctuations in biomass. Our biomass data contained zero and continuous positive values, therefore we used a Tweedie observation model [52, 53] in all models with a log link (Supporting information).

We chose covariates that represent certain features of the coastline and the *in situ* environment and are known to influence euphausiid distribution [16, 25, 32]. Depth and slope of the seafloor highlight the complex bathymetry of the region, including the three sea valleys of Queen Charlotte Sound and the Juan de Fuca Canyon system, and shallower regions on the Dixon Shelf (Fig 1). Distance to the coast and distance to the shelf edge (1000m isobar) combine to describe the location of land and the continental slope, representing the boundary between shelf waters and the open Pacific Ocean, as well as various sea canyons along the outer edge of the shelf (see S3A–S3D Fig). Distance to the 200m isobar and rugosity were also tested but omitted due to collinearity issues. These datasets were produced by the Marine Spatial Ecology Section, Fisheries and Oceans Canada, Nanaimo, BC [54]. Mean monthly sea surface temperature (SST, °C) [55] and mean monthly surface Chlorophyll A (mg/m^3^) [56] were obtained from online NOAA databases for April-September (averaged across 2012 to 2019) and were included to represent changes in the physical environment that occur through the spring transition and summer period when upwelling is strongest (see S3E and S3F Fig).

Global models, including all covariates (see Table 1) were used to highlight species-specific responses to environmental variables. For the larval model only, we included the local biomass of *E*. *pacifica* and *T*. *spinifera* adults as predictors as log(x +1). We standardized covariates by subtracting their mean and scaling by their standard deviation. This helped avoid computational issues when fitting the models and made the magnitude of the coefficients comparable. Depth and Chlorophyll A were log-transformed prior to this process to decrease the importance of large and small biomass values. Each variable was fit in the model as a spline. The number of basis functions (k) for each spline was selected based on the 95% confidence interval of the coefficient estimates that did not overlap zero and inspection of the model residual plots. Model fit was checked via residual plots. Covariates were included as fixed effects in all models, while separate spatial random fields were estimated for each month to allow seasonal (spring and summer) changes to be approximated. Final model formulas can be found in Table 1 and observed vs. predicted biomass plots can be viewed in S5 Fig. The shape of the relationship between each covariate and euphausiid biomass was further assessed to confirm that all relationships appeared biologically reasonable via conditional effects plots. We calculated conditional effect predictions and standard errors for each covariate while fixing the other covariates at the average value of conditions within the occupied depth range of each species (an average weighted by biomass found at each sampling location rather than the overall mean of all sample locations that is represented by zero for scaled variables). This made the illustrated conditional effects representative of abundance under realistic environmental conditions for each species.

**Table 1:** Model formulas, using pseudo-R code, for *E*. *pacifica*, *T*. *spinifera* and euphausiid larvae, including null model, depth- only model and full covariate model after covariate selection.

Group	Level	Predictors
Adult models	Null model	s(month, k = 6) + as.factor(year)
(*E*. *Pacifica* and *T*.	Depth-only	s(month, k = 6) + as.factor(year) + s(depth, k = 3)
*spinifera*)	Full model	s(month, k = 6) + as.factor(year) + s(depth, k = 3) + s(distance to coast, k = 4) + s(distance to 1000m isobar, k = 3) + s(slope, k = 4) + s(mean monthly SST, k = 3) + s(mean monthly chlorophyll, k = 3)
Larvae	Null model	s(month, k = 6) + as.factor(year)
	Depth-only	s(month, k = 6) + as.factor(year) + s(depth, k = 3)
	Full model	As above + s(*E. pacifica*, k = 4) + s(*T. spinifera*, k = 4)

#### Identification and seasonality of biomass hotspots

To investigate seasonal variability in the spatial distribution of *E*. *pacifica*, *T*. *spinifera* and euphausiid larvae on the BC coast, we generated maps of mean predicted biomass (mg/m^2^) for each of the six months. We did not predict for April in the northern bioregion (see Fig 1) as we had no observed data points in this region. Predictions of the biomass of *E*. *pacifica*, *T*. *spinifera*, and euphausiid larvae were based on all fixed and random effects for each month along a 3km^2^ grid. We also calculated the spatial ratio of E:T biomass (*E*. *pacifica* / *T*. *spinifera*), to highlight regions where one species dominated and regions that were preferred habitat for both species.

A major aim of this work was to assess if hotspots of the three euphausiid groups modelled were persistent through the important spring and summer period. To accomplish this, we first determined the spatial coherence/patchiness of predicted euphausiid biomass by employing the Getis-Ord statistic [*Gi;* 57]. *Gi* is a statistical Z-score of local clustering (or spatial intensity) relative to the background spatial mean and standard deviation, and therefore is a quantitative measure of hotspot distribution and intensity [see Refs 13, 58, 59 for practical application of the method]. Moran’s *I* test for spatial autocorrelation was used to inform the spatial neighborhoods (*d*, distance matrix defining the distance between cells to be considered as ‘neighbours’), which was set at 10km. Separate *Gi* tests were run for each of the six months for each of the three groups of modelled euphausiids. Z-scores were calculated for the same 3km^2^ grid as model predictions and mapped. Moran’s *I* tests and *Gi* tests were run using ‘spdep’ [60] and ‘usdm’ [61] R packages, respectively. We also combined biomass of the two adult species (‘total adults’) to map combined hotspots of *E*. *pacifica* and *T*. *spinifera*, which make up the majority of biomass on the BC coast.

To characterize the distribution and intensity of biomass hotspots for each euphausiid group, we mapped the 90^th^ and 95^th^ (top 10% and top 5%) of Z-scores. Percentiles were calculated across the 6-month period for each species and larvae to allow comparisons between spring and summer periods (i.e., to highlight temporal peaks in hotspots for each group). To categorise the persistence of hotspots, we counted the number of times a cell fell in the 95^th^ percentile across months, giving each hotspot cell a score of 1–6. Cells that were hotspots through each month had a perfect score of 6 and were considered persistent. Finally, we calculated the area (km^2^) of hotspots, and persistent hotspots, for each of the oceanographic ecosections in Fig 1.

## Results

### Model fit and selection

**Fig 2:**
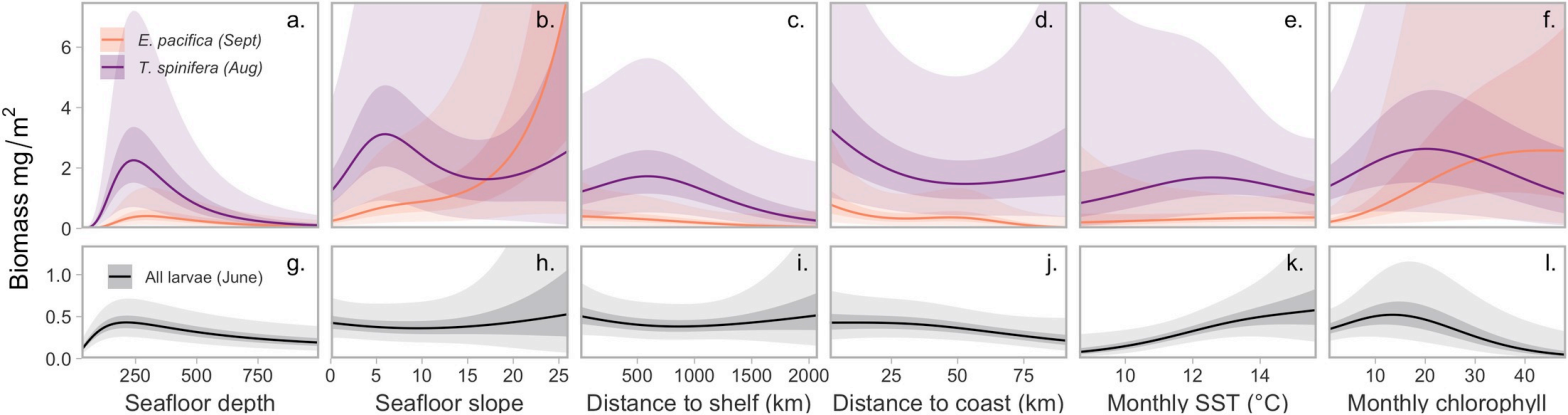
**Conditional effects of each of the fixed effects (x-axis) included in the full models, illustrating differences in peak biomass density (y-axis) between species (top row) and for total larval biomass (bottom row).** The height of each curve represents the predicted density in the month with the highest estimated biomass densities for each group, at the weighted mean occupied conditions of all other covariates, and at the mean of spatiotemporal modelled effects. The shapes of these curves were not allowed to interact with other covariates. Lines represent means and shaded ribbons represent 50% CIs, with the most lightly shaded area representing 95% CIs.

The inclusion of depth as a predictor reduced the standard deviation of the spatiotemporal random fields (*σ_ϵ_*; see S1 File for equations) in all models (Table 2). For all three groups, conditional-effect plots indicated that the shape of this depth-biomass relationship was roughly quadratic (Fig 2A). The addition of more geomorphic and dynamic covariates reduced the magnitude of this spatiotemporal random field standard deviation *σ_ϵ_* in two out of three models (full models—Table 2), with the largest reduction for euphausiid larvae. *σ_ϵ_* compared with the depth only model for *T*. *spinifera* (Table 2); however, the AIC and residual plots (not shown) indicated that the full model was a more parsimonious model fit. The larval model showed a substantial decrease in AIC between the depth-only model and the full covariate model (ΔAIC 130, Table 2). For larvae, the additional inclusion of adult distribution predictors led to an improvement in capturing spatiotemporal variability and therefore we included these in the final model. Based on AIC and inspection of residuals, the rest of the results will focus on analysing the full covariate models for all euphausiids.

**Table 2:** Model selection characteristics for *E. pacifica, T. spinifera,* and euphausiid larvae including degrees of freedom (df), AIC from best model in parentheses and spatiotemporal random field standard deviation (*σ_ϵ_*).

Group	Model	df	AIC	*σ_ϵ_*	Lower	Upper
					CI	CI
*E*. *pacifica*	Just Time	37	2266	2.64	2.17	3.21
	Time and depth	39	2174	1.63	1.30	2.04
	Full model	51	2159	1.39	1.09	1.78
*T*. *spinifera*	Just time	37	3138	2.46	2.01	3.00
	Time and depth	39	3024	1.26	0.99	1.61
	Full model	50	3020	1.54	0.90	2.63
Larvae	Just time	37	-2467	0.89	0.74	1.07
	Time and depth	39	-2513	0.77	0.63	0.95
	Full model	55	-2643	0.59	0.46	0.74

Confidence intervals (CIs) are at the 95% level.

Conditional-effect plot responses were not scaled to represent relative influence of predictors for each model, and therefore represent actual predicted biomass of the euphausiid groups. Fig 2 indicated a tendency for *E*. *pacifica* biomass to be highest in areas where the edge of the continental slope described here as the 1000m isobar coincided with the coast (see Fig 1 and S3C and S3D Fig). This environment is found in the north of the study region, to the west of Haida Gwaii, where the continental shelf is narrow. *E*. *pacifica* biomass was also highest in areas of increased seafloor slope, and during months with high chlorophyll. Conditional-effect plots indicated the relationship between *T*. *spinifera* biomass and the majority of covariates tested fit a quadratic shape. Relationships were weak but indicated the highest biomass of this species occurred nearer to the coast, further from the shelf, and in flatter areas than *E*. *pacifica*. Euphausiid larvae biomass was positively correlated with both adult biomass; however, this relationship was much stronger for *T*. *spinifera* than *E*. *pacifica* biomass (see S4 Fig for conditional effects plot). Larval biomass was also significantly linked to monthly means of the dynamic variables tested; there was a positive relationship between larval biomass and both monthly chlorophyll and SST (Fig 2).

### Predicted spatiotemporal biomass variability and persistence of euphausiid hotspots

**Fig 3:**
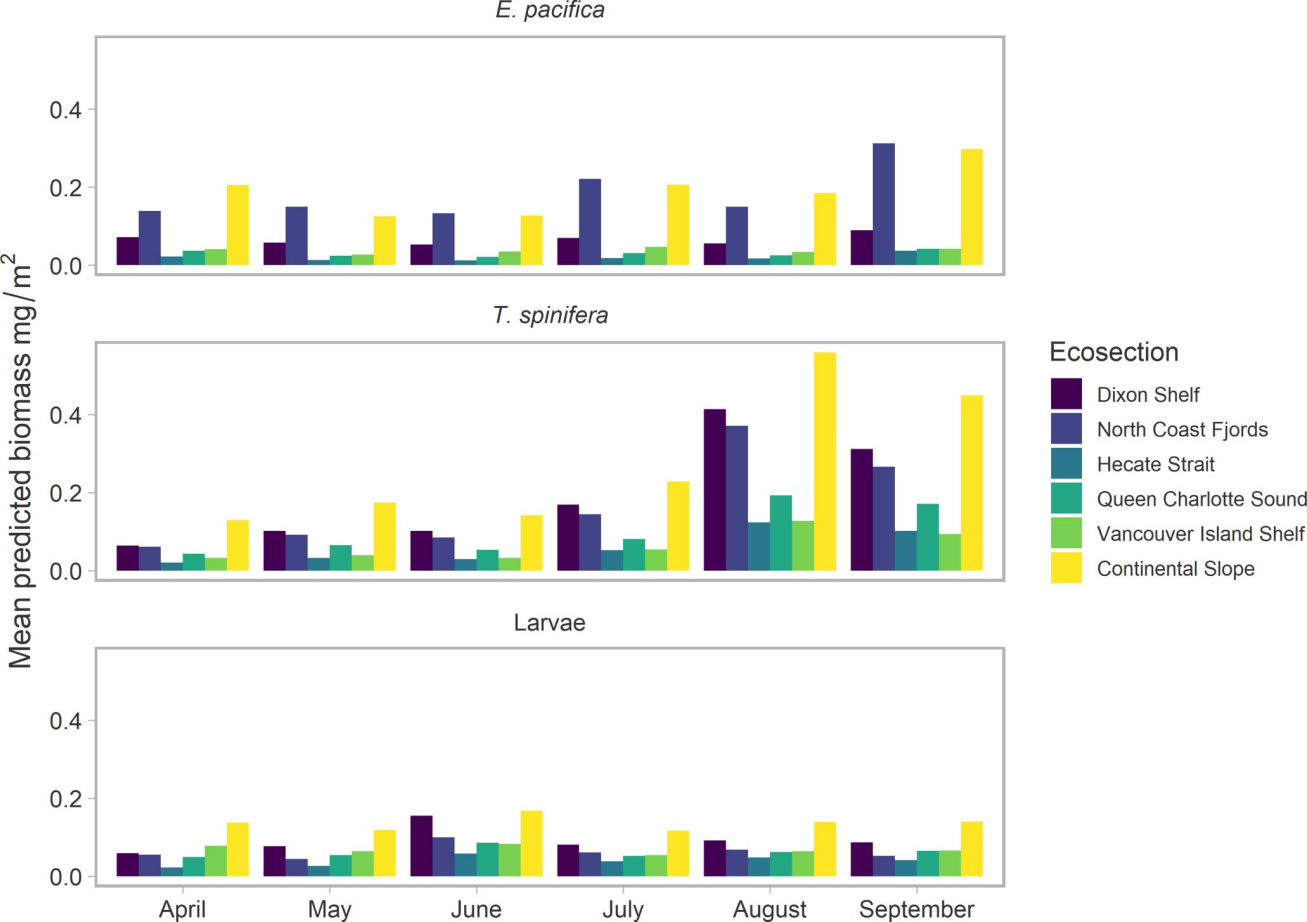
Mean predicted biomass mg/m^2^ for each month across the prediction grid, calculated for each oceanographic ecosection displayed in Fig 1.

**Fig 4:**
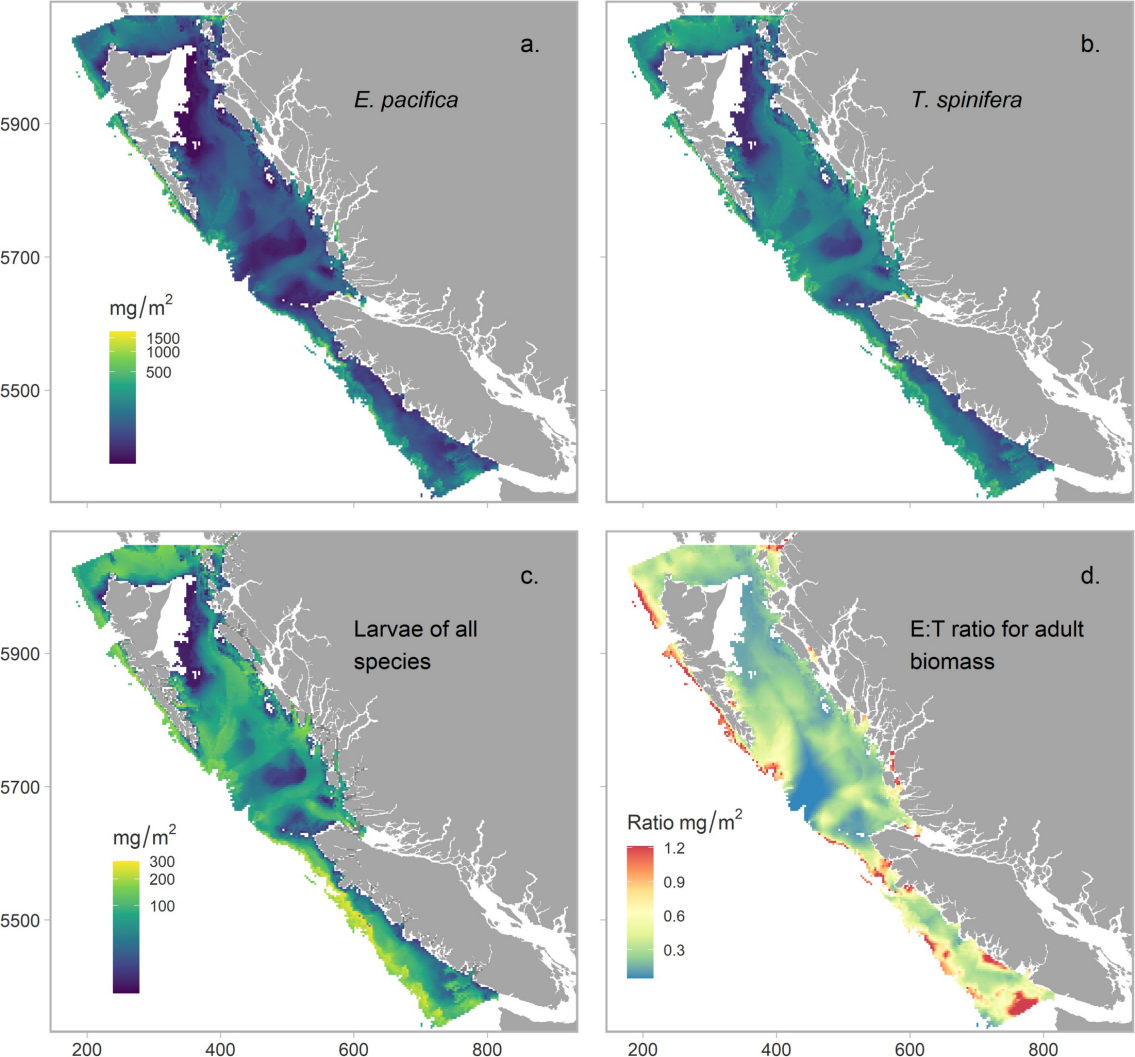
Mean predicted biomass from each model for a. *E*. *pacifica*, b. *T spinifera*, c. euphausiid larvae averaged across spring and summer (April-September). d. The spatial distribution of the predicted ratio of *E*. *pacific*a:*T*. *spinifera* biomass. All predictions were made across a 3km^2^ prediction grid to 1000m depth. Mean calculations did not include predictions for April in the northern bioregion due to gaps in the observed data.

Seasonal biomass fluctuations were well represented by each model for the different groups. Mean predicted biomass of adults was similar, and low throughout spring, until August and September when *T*. *spinifera* exhibited a large peak in biomass (Fig 3). *T*. *spinifera* biomass increased across all regions during these months, but was particularly apparent on the Dixon Shelf, the North Coast Fjords, and the Continental slope (Fig 3). Predicated *E*. *pacifica* biomass exhibited a smaller and slightly later peak in September, which was mostly confined to the North Coast Fjords and along the Continental slope. This spatial pattern is also apparent in Fig 4A and 4B, with consistently higher biomass of *T*. *spinifera* across all regions in the study area, whereas higher *E*. *pacifica* biomass was only concentrated along the Continental slope. Overall, euphausiid larvae biomass was smaller than the two adults (Fig 4C) and rose through the spring to peak in June before decreasing slightly (Fig 3). A comparison of the observed vs. predicted biomass from each model can be found in the (S5 Fig).

**Fig 5:**
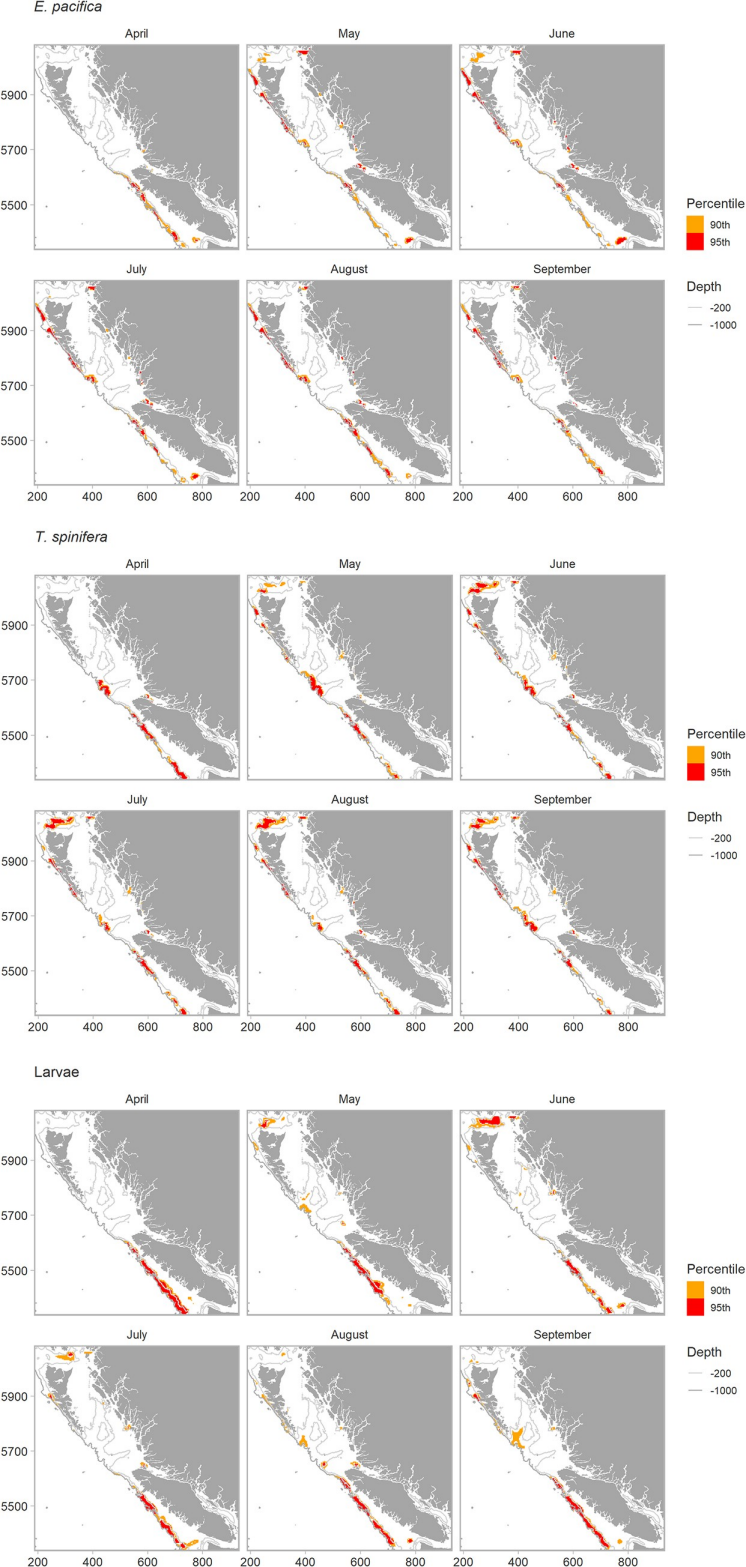
Predicted distribution of *Gi* Z-scores in the 90^th^ (orange) and 95^th^ (red) percentile for (a) *E*. *pacifica*, (b) *T*. *spinifera*, and (c) euphausiid larvae. For May to September, gaps off the west coast of Haida Gwaii indicate gaps in predictor surfaces (NAs), we did not predict the distribution of hotshots in the northern bioregion for April due to gaps in the observed data (see S6 Fig).

**Fig 6:**
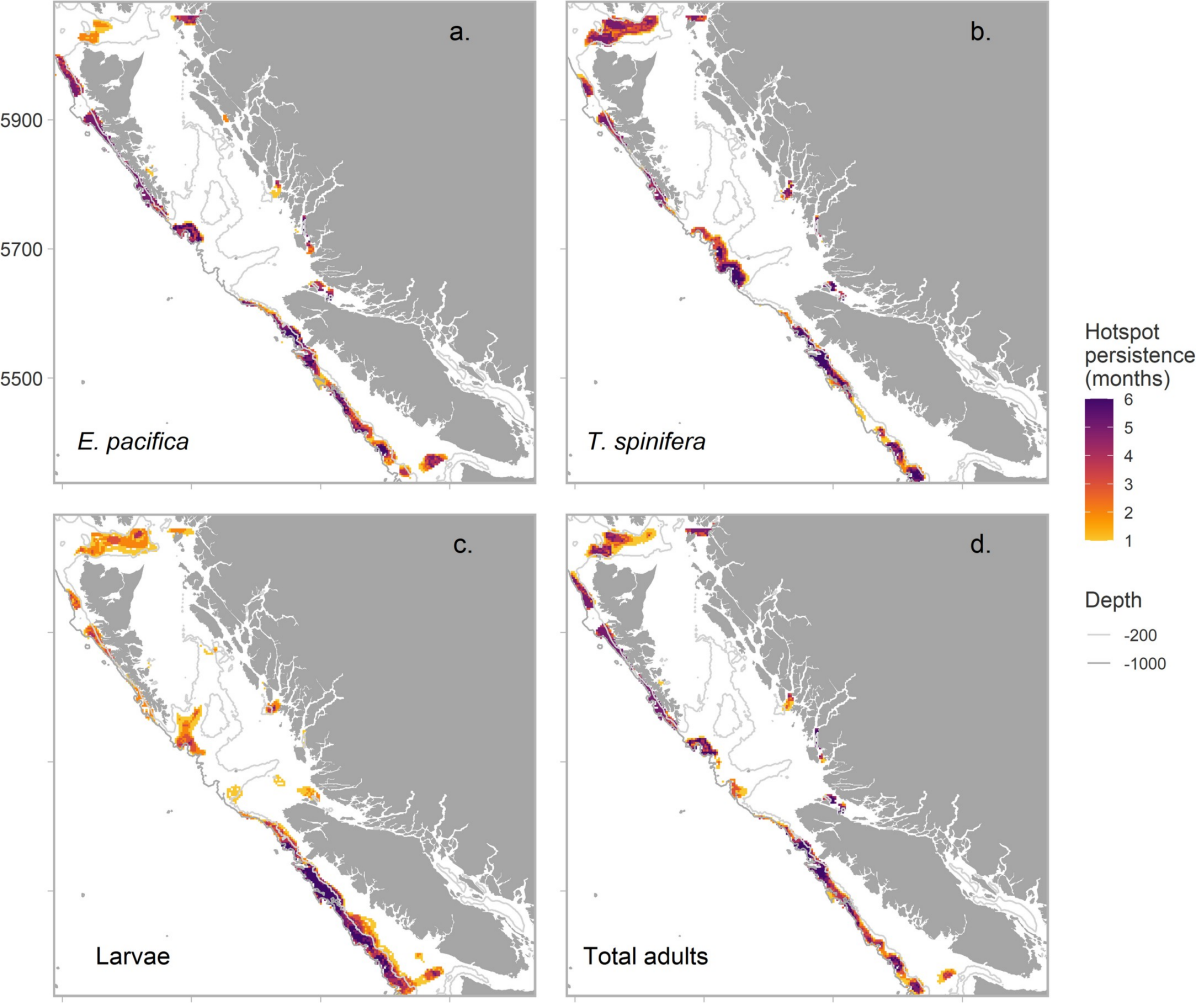
Persistence of 90^th^ percentile of Z-scores, or hotspots, over the 6-month period examined. 1 = grid cell counted as a hotspot only in one month, 6 = grid cell persistently ‘hot’ in all 6 months, for (a) *E*. *pacifica*, (b) *T*. *spinifera*, (c) euphausiid larvae and, (d) total euphausiid adults (*E*. *pacifica* + *T*. *spinifera*).

Mapped Z-scores used to calculate hotspots for each group can be found in the, S6A–S6C Fig. All three groups exhibited some seasonal variability in the distribution of hotspots within the 90^th^ percentile across months (Fig 5A–5C); however, larval hotspots exhibited the least persistence (Fig 6A–6C). For larvae, the only persistent hotspot area (classified as present across all 6 months) was found along the west coast of Vancouver Island (or the southern part of the Continental slope ecosection); therefore, the only common persistent hotspot between all three groups was this area. (Fig 6A–6C). For adults, there was high commonality in predicted hotspot distribution, with persistent hotspots along the Continental slope and the fjords of the Northern Coast. Differences between the two species occurred on the Dixon shelf, where *T*. *spinifera* exhibited a large and persistent hotspot, whereas *E*. *pacifica* did not, and within the Juan de Fuca Eddy system, which was a hotspot region for *E*. *pacifica* but never for *T*. *spinifera*.

Overall adult hotspots displayed higher persistence than larvae, particularly along the along the Continental slope (Fig 6A and 6B and Table 3), with *E*. *pacifica* hotspots exhibiting the highest persistence by area. Hotspots in the 95^th^ percentile for *E*. *pacifica* and euphausiid larvae encompassed a similar area for all three groups; however, for larvae this area was contained along the west coast of Vancouver Island, with few other hotspot areas in other regions around the coast (Table 3; Fig 6). Despite the overall predicted biomass of larvae being lower than for the two adults (Fig 4), hotspots of this group covered the largest area (Fig 6C and Table 3), indicating that larval biomass was patchier, with higher levels of spatial disparity than adult biomass. The intensity of hotspots (Z-score), or clustered biomass within hotspots, was also highest for larvae (Table 3).

**Table 3:** Summary of hotspot intensity and persistence for *E*. *pacifica*, *T*. *spinifera*, and euphausiid larvae.

Region	Type and area of hotspot km^2^	*E*. *pacifica*	*T*. *spinifera*	Larvae
Dixon Shelf	Total (km^2^)	822	2178	2550
	Persistent (km^2^)	66	222	0
	Month of peak Z-score	May (16.9)	Aug (13.2)	June (14.1)
Vancouver Island Shelf	Total	1050	240	2478
	Persistent	168	72	216
	Month of peak Z-score	June (19.9)	April (15.1)	April (18.9)
Hecate Strait	Total	108	0	24
	Persistent	0	0	0
	Month of peak Z-score	April (10.1)	0	Aug (6.3)
North coastal fjords	Total	384	186	168
	Persistent	234	66	0
	Month of peak Z-score	June (16.5)	Aug (9.2)	June (9.2)
Continental slope	Total	5466	5484	5688
	Persistent	**3180**	2340	1848
	Month of peak Z-score	April (20.9)	April (19.6)	April (26.2)
	*North of 555 km north*	3168	3240	2736
	Total			
	Persistent	**2478**	1386	132
	Month of peak Z-score	April (20.9)	May (15.9)	Aug (14.2)
	*South of 555 km north*			
	Total	2298	2244	2952
	Persistent	702	954	1716
	Month of peak Z-score	Aug (14.8)	April (19.6)	April (26.2)
Queen Charlotte	Total	372	240	1392
Sound	Persistent	42	42	0
	Month of peak Z-score	Sept (15.8)	June (8.0)	June (9.8)
Total BC coast	Total	8202	8328	12300
	Persistent	**3690**	2742	2064
	Month of peak Z-score	April (20.9)	April (19.6)	April (26.2)
	Month of largest hotspot (95^th^ percentile)	April (3330 km^2^)	August (2910)	April (3450)

Includes the area (km^2^) of the total (levels 1–6, Fig 6) and persistent (level 6, Fig 6) hotspot for each species for each spatial region in Fig 1. The month and value of the highest Z-score present in each region is also shown. The highest value for each metric is presented in bold. The continental slope ecosection is further divided using boundaries for the north and south bioregion areas (see Fig 1 for division). Alternative summary statistics are present at the bottom of the table; the month of peak hotspot intensity is characterized as the month with the largest hotspot area in the 99^th^ percentile for the whole coast.

The area (km^2^) of predicted hotspots showed high spatial variability for all three groups modelled. As well as exhibiting the most persistent hotspots, the Continental slope was also the region with the largest hotspots for all three groups (Table 3). However, there were differences between the northern and the southern slope regions (see Fig 1 for bioregion divisions). Hotspots of both adult species were larger and more persistent along the northern slope, off the coast of Haida Gwaii, and along the edge of Queen Charlotte Sound, while larval hotspots were larger and more persistent in the south, along the continental slope off the west coast of Vancouver Island (Table 3, Fig 6C).

We hypothesized that the Juan de Fuca Eddy system would be a hotspot region for euphausiids (see Fig 1 for location). However, this region was not predicted to be a persistent hotspot area for any of the euphausiids. Hotspots here only occurred for *E*. *pacifica* and larvae, and were temporally patchy (Fig 6A and 6C). We also hypothesized that the three sea valleys of Hecate Strait would be important hotspot areas due to increased seafloor complexity. Persistent hotspots occurred for both *E*. *pacifica* and *T*. *spinifera* along the outer edges of these sea valleys, where the valleys deepened to canyons along the edge of the continental shelf. *E*. *pacifica* and *T*. *spinifera* hotspots were associated with different valleys however (see ratio of E.T biomass, Fig 4D). Larvae also exhibited hotspots along these sea valleys, but they were unpersistent and occurred in shallower areas on the shelf than adult hotspots. Larval hotspots only occurred in these areas in May, August and September (Fig 5C).

## Discussion

We used spatiotemporal modeling of more than 27 years of net-capture data to identify regions of high euphausiid biomass, or ‘hotspots’, for the first time along the Pacific coast of Canada. In addition, few studies have characterized species-specific hotspots of euphausiids. This work is the first step towards quantifying the physical and climate variables that lead to the development and persistence of euphausiid hotspots, with the ultimate goal of delineating important foraging regions for euphausiid vertebrate predators along the BC coast. This type of information is critical, but often lacking, for the development of conservation management strategies such as marine spatial planning [62–64]. However, as with any modelling exercise, some caution should be exercised when interpreting the results. For instance, the modelling framework explained a considerable proportion of variation through spatiotemporal random effects. In addition, as our focus was on seasonal changes, phenological differences between years such as differences in the timing of development of larval biomass, were not considered. Future work should focus on quantifying the effect of these interannual phenological changes, and on improving links between the spatial and temporal scale of response variables with environmental predictors. Both of these factors would likely improve model fit, and may result in tighter coupling between environmental variables such as temperature and the response, which almost certainly shaped euphausiid distribution, but for which we only found a strong correlation with larvae.

### Spatiotemporal variability in biomass peaks

In both the observed and modelled data, biomass of *E*. *pacifica* and *T*. *spinifera* rose throughout the spring to peak in September and August respectively (see S7 Fig). We can infer spawning of both species occurred around the spring transition in April/May, as both *E*. *pacifica* and *T*. *spinifera* are thought to reach lengths of >10 mm (considered here to be adults) 2–4 months after spawning [2, 12, 29]. In both the northern and central CCE (Oregon and California), reproduction in *E*. *pacifica* has been linked to the onset of upwelling (usually April-June) [2, 28], which would also coincide with our observed peaks in euphausiid larvae along the coast in June.

Tanasichuk [12, 29] conducted an in-depth study of the growth and reproduction of *E*. *pacifica* and *T*. *spinifera* in Barkley Sound, an inshore area along the west coast of Vancouver Island. During 1991–1994, abundances of larger size classes mainly occurred May to September for *T*. *spinifera*, and May to July for *E*. *pacifica;* however, there was large interannual variability (Tanasichuk 1998a, b [12, 29]). We found that the peaks in biomass that lead to hotspots of both species were patchily distributed, both spatially and across months. These differences increase the potential for species-specific hotspots to occur, effecting the availability of these two dominant species for euphausiid predators.

In many cases, knowledge of the alongshore distribution of euphausiid hotspots comes from acoustic studies, which are unable to differentiate between species. *T*. *spinifera* are larger and have a higher lipid content than *E*. *pacifica* [65], and some predators preferentially select this species during chick rearing. Cassin’s auklets in the CCE have been found to feed on *E*. *pacifica* during pre-breeding and the early part of the breeding season, and then to switch to more energy rich *T*. *spinifera* during chick-rearing [1]. In addition, chick growth rates were positively correlated with the proportion of *T*. *spinifera* in auklet diets [11]. This suggests that species-specific hotspots of euphausiids may be important for breeding success in seabird predators. Whales can also be selective foragers; a recent study in southern California found that blue whales primarily selected *T*. *spinifera*, even when other euphausiid species were present [66].

### The spatiotemporal distribution, intensity and persistence of hotspots

In agreement with studies further south in the CCE, our models indicate that euphausiid hotspots were orientated alongshore in areas associated with the shelf break and specific depth contours [14–16]. This study adds to the large body of literature that suggests that a combination of depth, and the edges that occur along topographic features such as the continental slope and seafloor valleys and canyons, are unequivocally important to the development of areas of higher euphausiid biomass [15, 25, 67–69]. We found similarities between the overall distribution of adults, and hotspots of both species were highest over the continental slope, particularly off the west coast of Haida Gwaii. Larger differences may have been observed between the distribution of these two species in waters deeper than 1000m [14], however, unfortunately sampling points in deeper waters were too sparse to be included in our models.

We identified a persistent hotspot region for both adult species and larvae along the shelf break on the west coast of Vancouver Island, which was the only common persistent region between groups through the spring and summer months. This region includes the highly productive La Perouse bank region (south-west Vancouver Island) and is well studied, being one of the most productive fishing grounds off western North America [42]. The southern coast of Vancouver Island is part of the upwelling California Current Ecosystem (CCE), and is highly productive following the spring transition, particularly within the Juan de Fuca Eddy system. Previous studies found that the eddy system was important in generating the biomass of euphausiids required by key euphausiid predators during the summer, particularly Pacific Hake [70, 71]. The northern end of Vancouver Island is part of the down-welling dominated Alaska current, and is considered less productive than the southern section [72]. However, this work highlights the importance of this northern region to euphausiid hotspot development, with persistent hotspots for both adults found here (Fig 6B and 6C).

Mackas et al. [31] suggested a model for euphausiid transport in this region related to local current patterns, euphausiid diel migration, and upwelling. They hypothesized that euphausiid biomass peaks at the shelf break occurred just below, or just inside, upwelling currents converging upwards along the continental slope, optimizing their food supply. Euphausiids avoid advection during the day and night using the surface California Current and subsurface California Current Undercurrent. They do this by positioning at the inshore margin of the shelf break current where equatorward currents are weak. Therefore, at night, they minimize surface (and offshore) advection and are transported onshore and slightly poleward during the day. In this way they maintain the same general position in the water column. This was supported by the results of Lu et al. [3] in the same area, and by Swartzman et al. [73], further south in the CCE. Due to these differences in surface and deep current directions, and the breadth of the continental slope, high production on the shelf and at the shelf-break from local productivity and during large scale upwelling events is extended over a large latitudinal range. We hypothesize that this mechanism may be partly responsible for the high persistence of euphausiid hotspots along the shelf.

A similar process may be responsible for hotspots on the shelf of Queen Charlotte Sound. The shelf encompasses three sea-troughs of ~300 m depth, and multiple sub-marine canyons at the shelf edge [74]. Sea valleys and canyons disrupt alongshore current flow, leading to areas of retention at daytime distributional depths of euphausiids [15, 16]. In addition, these troughs can act as conduits for oceanic waters during upwelling [75], so are often areas of high productivity [27, 76]. They present an ideal habitat for euphausiid larval development [15, 25], and were predicted areas of larval hotspots in this study. In upwelling systems, an optimum window for euphausiid hotspot development has been proposed that balances advective upwelling processes with retention [16, 77]. Canyons are considered regions of high importance to the development of euphausiid hotspots further south in the CCE [15], and were persistent hotspot regions for both adults.

Larval hotspots covered more area, and exhibited higher intensity than adult hotspots, despite overall biomass being lower, indicating tight spatial clustering of biomass. The persistence of larval hotspots was generally low apart from the west coast of Vancouver Island. This is consistent with previous observed patterns for this region [3] and in other regions [78]. Aggregations of euphausiid larval would be expected to be more dynamic than adult hotspots; larvae are less motile than adults and therefore, do not perform extended vertical diel migration. During summer upwelling on the west coast of Vancouver Island, larvae are advected seaward and equatorward in the top 10-30m of the water column as they develop, until the depth range of their diel vertical migration becomes deep enough that they are less exposed to surface seaward-currents [31]. Larval biomass is therefore likely to be more patchily distributed across the coast than adult biomass, perhaps with the majority of biomass deposited at the seaward edge of upwelling cells. However, there is large variability associated with upwelling current patterns, causing differences in the distribution of larval biomass between years, and perhaps leading to the predicted larger spatial area of hotspots averaged across these years. Lu et al. [3], compared larval distribution between two years along the west coast of Vancouver Island, and found differences in the distribution of larvae with respect to the coast between years due to an El Niño event. Further south off Oregon, the same El Niño event caused an increase in the coastal abundance of larvae due to strong onshore advection, ensuring larvae were retained in production centers near to the coast [78].

The Juan de Fuca Eddy and canyon system (see Fig 1), is an area of seasonally heightened primary productivity compared with surrounding water, and was therefore expected to be an area of importance for euphausiids [15, 32]. Hotspots in the 95^th^ percentile occurred in the eddy for *E*. *pacifica* and larvae from June to August, however, and never for *T*. *spinifera*. Eddy systems enhance mixing and productivity in surface waters, and entrain less motile larvae and other plankton within the eddy structure, leading to higher larval and adult survival [79]. Predation pressure can account for the temporally patchy occurrence of euphausiid hotspots in the eddy during mid-summer. Robinson et al. [80] attributed the rapid decline in euphausiid biomass over the mid-summer period to predation by Pacific Hake. This commercial fish predator migrates onto the southern BC continental shelf in June and are heavy consumers of euphausiids [81]. However, the lack of high biomass of *T*. *spinifera* requires further investigation.

Areas of topographic complexity, which are important to euphausiids, are also hotspots for euphausiid predators [6, 21, 26, 82, 83]. The persistence of euphausiid hotspots will aid in the predictability of important foraging regions for predators, which is critical information for management strategies such as marine spatial planning. In an ecological sense, if hotspots of prey are persistent through high and low prey availability, as hotspots along the Continental slope are predicted to be, these regions will be important for ecosystem resilience through extreme events such as El Niño and marine heatwaves. Future work could focus on characterising the interannual persistence of these hotspots, and other productive euphausiid regions on the BC coast such as Dixon shelf and the west coast of Haida Gwaii. Given use of these areas for foraging by species of conservation concern—including Eulachon, Pacific Hake, Pacific Herring, salmon species, and seabirds [21, 31, 84–86]—this would contribute important information for ecosystem-based management of these coastal regions.

## Supporting information

S1 File Supplementary methods. (DOCX)

S1 Fig SPDE mesh used in euphausiid modelling for the British Columbia coast, shelf and slope. Red dots represent knot locations (n = 200) and open black circles represent the locations of euphausiid net-hauls. Triangles represent the SPDE mesh.
(PDF)

S2 Fig Distribution of euphausiid sampling stations as modelled. (PDF)

S3 Fig a-f. Model covariates included in all full models. a-d show static, geomorphic covariates (Depth, Slope, Distance to coast (km), Distance to shelf or 1000m isobar (km). e and f show the dynamic (time-varying) variables: monthly mean of chlorophyll and sea surface temperature (SST).
(TIF)

S4 Fig Conditional effects of adult distribution (fixed effects, x-axis) on larval biomass (y-axis). See Fig 2 for full caption.
(TIF)

S5 Fig Observed vs. predicted plots for (a) *E*. *pacifica*, (b) *T*. *spinifera* and, (c) euphausiid larvae from the full covariate model specified in Table 1. Vertical lines of dots represent values of 0 at an arbitrary location on the left edge of the y-axis.
(TIF)

S6 Fig a-c. Spatiotemporal variability in Z-score from Getis Ord hotspot analysis for April to September for (a) *E pacifica*, (b) *T*. *spinifera*, and (c) Euphausiid larvae. Predictions were not made for April due to gaps in the observed data.
(TIF)

S7 Fig Seasonal comparison in mean observed vs. mean model estimates of biomass for each of the three models for *E*. *pacifica*, *T*. *spinifera*, and euphausiid larvae (left to right).
(TIF)
