# Supplementary material for: Factors affecting the seasonal distribution and biomass of E. pacifica and T. spinifera along the Pacific coast of Canada: A spatiotemporal modelling approach
Source: PLoS One. 2021 May 14;16(5):e0249818. doi: 10.1371/journal.pone.0249818 (PMC8121349; doi:10.1371/journal.pone.0249818)
Supplement: S1 File — (DOCX) [file pone.0249818.s001.docx]

**Supplementary Methods**

As outlined in the main text, models are geostatistical and based on a stochastic partial differential equations (SPDE) mesh. The mesh generated for these models included 200 knots for all models, including null, depth-time only and full models (see Table 1). The general model form can be represented as

$$\begin{matrix} y_{s,t} & \sim\text{Tweedie}\left( \mu_{s,t},p,\phi\right), 1<p<2 , \\ \mu_{s,t} & =\text{exp}\left( \mathbf{X}_{s,t}\boldsymbol{\beta}+\epsilon_{s,m} \right), \\ & \\ \boldsymbol{\delta}_{m=1} & \sim\text{MVNormal}(\mathbf{0},\boldsymbol{\Sigma}_{\epsilon}), \\ \boldsymbol{\delta}_{m>1} & =\phi\boldsymbol{\delta}_{t-1}+\sqrt{1-\phi^{2}}\boldsymbol{\epsilon}_{m}, \boldsymbol{\epsilon}_{m}\sim\text{MVNormal}\left( \mathbf{0},\boldsymbol{\Sigma}_{\epsilon} \right), \end{matrix}$$

where $y_{s,t}$ represents the observed biomass density at a point in space $s$ and time $t$. The index $m$represents a month. The symbol $\mu$ represents the mean and $p$ and $\phi$ represent the Tweedie power and dispersion parameters. The symbol $\mathbf{X}_{s,t}$ represent a vector of predictors for that location in time and $\boldsymbol{\beta}$ represents a corresponding vector of coefficients; many of the coefficients correspond to basis functions from the smooth terms. The symbol $\boldsymbol{\epsilon}_{s,m}$ represent spatiotemporal random effects drawn from Gaussian Markov random fields with covariance matrices $\boldsymbol{\Sigma}_{\omega}$ and $\boldsymbol{\Sigma}_{\epsilon}$. The spatiotemporal random fields are allowed to follow a first order autoregressive structure (AR1) by month. The marginal standard deviation of $\epsilon_{s,m}$is defined as$\sigma_{\epsilon}$.
