# Supplementary figures and images for: Factors affecting the seasonal distribution and biomass of E. pacifica and T. spinifera along the Pacific coast of Canada: A spatiotemporal modelling approach
Source: PLoS One. 2021 May 14;16(5):e0249818. doi: 10.1371/journal.pone.0249818 (PMC8121349; doi:10.1371/journal.pone.0249818)

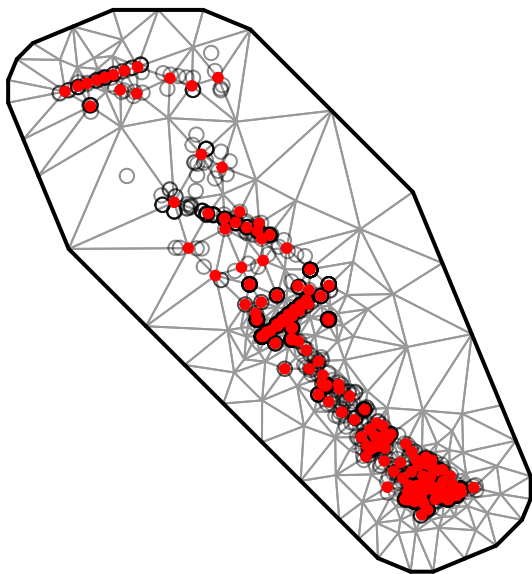

Supplement: S1 Fig — Red dots represent knot locations (n = 200) and open black circles represent the locations of euphausiid net-hauls. Triangles represent the SPDE mesh. (PDF) [file pone.0249818.s002.pdf]

April

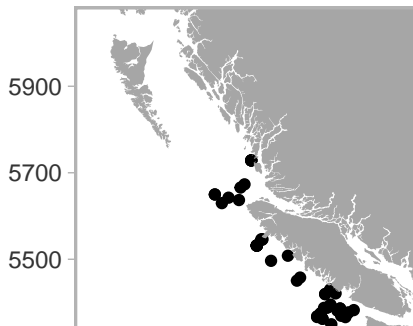

May

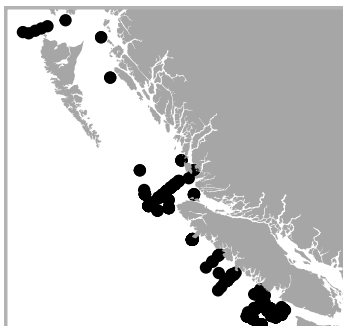

June

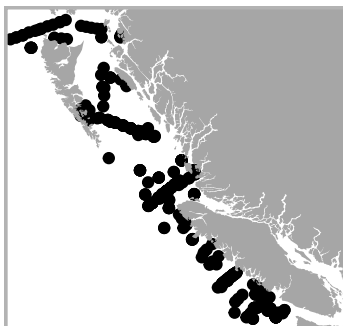

July

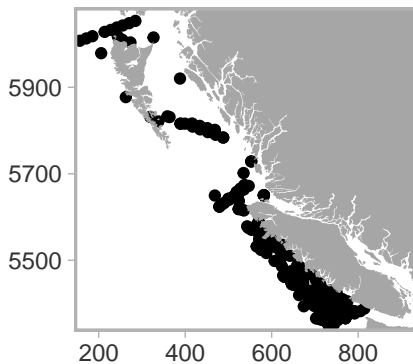

August

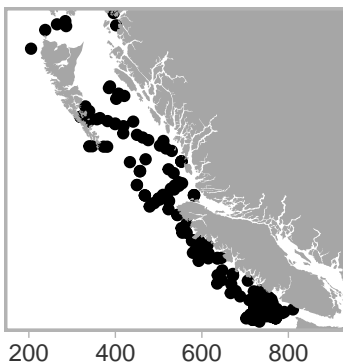

September

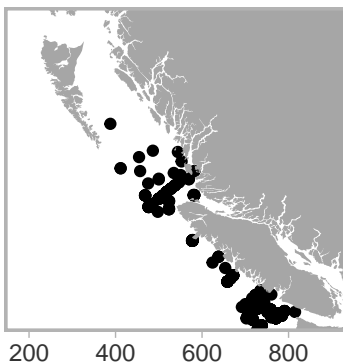

Supplement: S2 Fig — (PDF) [file pone.0249818.s003.pdf]

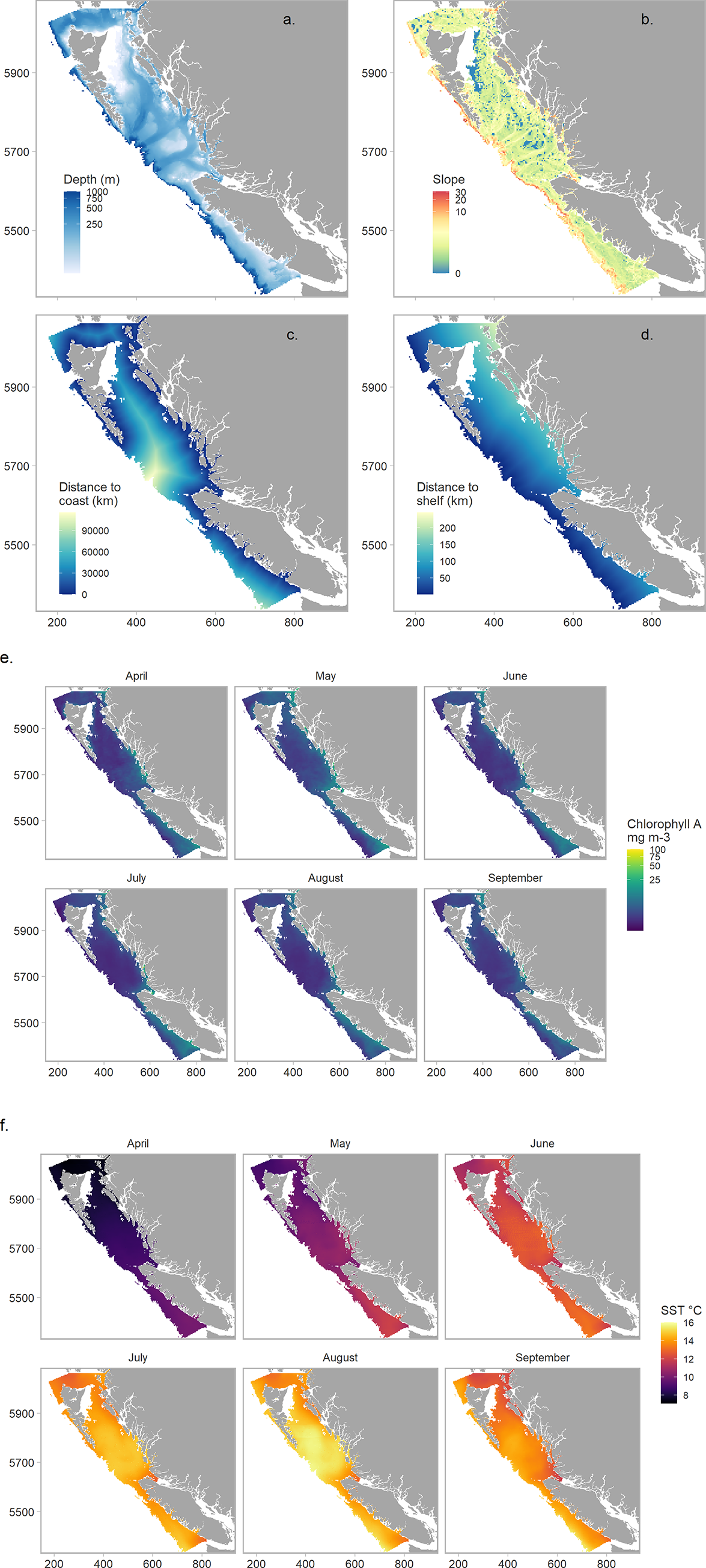

Supplement: S3 Fig — a-f. Model covariates included in all full models. a-d show static, geomorphic covariates (Depth, Slope, Distance to coast (km), Distance to shelf or 1000m isobar (km). e and f show the dynamic (time-varying) variables: monthly mean of chlorophyll and sea surface temperature (SST). (TIF) [file pone.0249818.s004.tif]

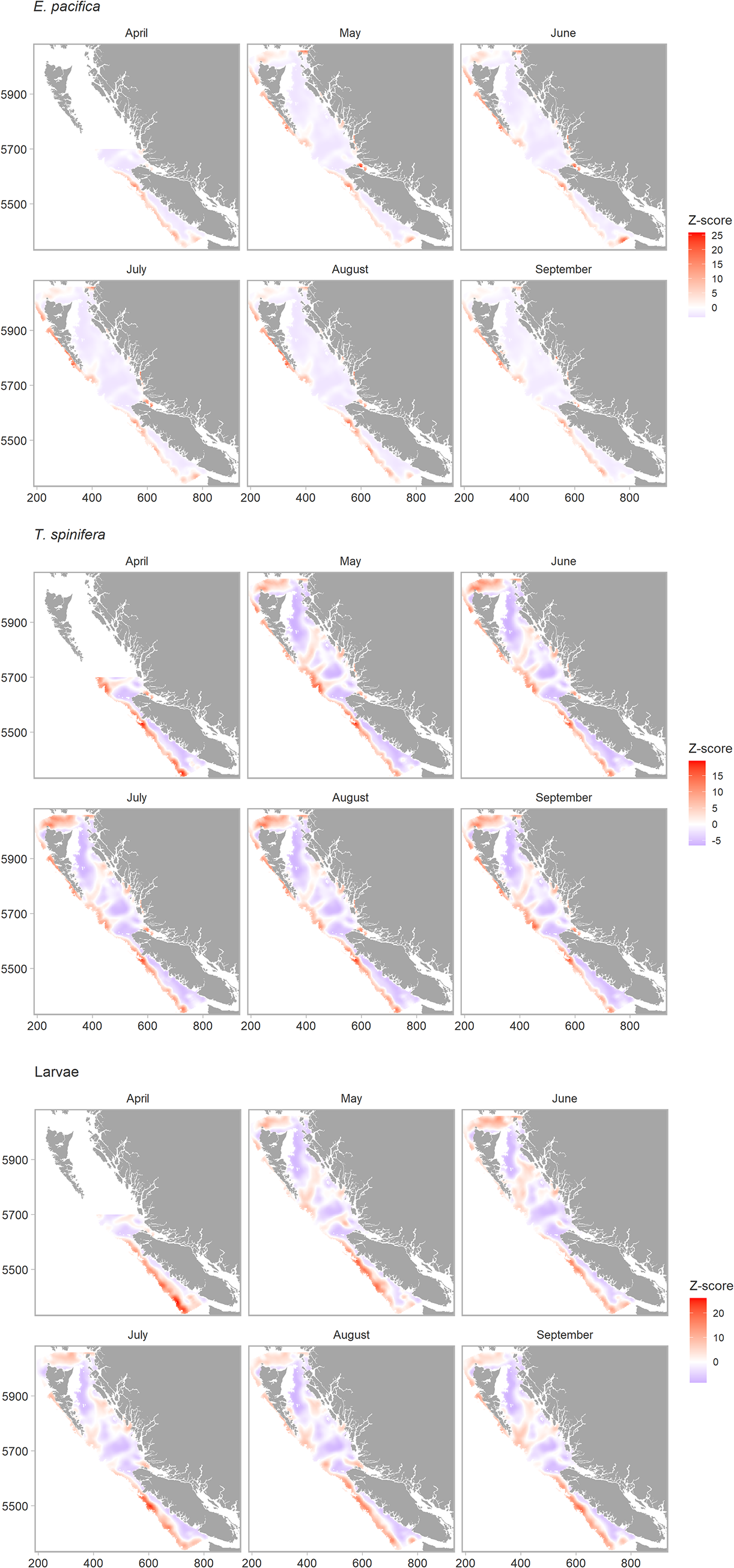

Supplement: S6 Fig — a-c. Spatiotemporal variability in Z-score from Getis Ord hotspot analysis for April to September for (a) E pacifica, (b) T. spinifera, and (c) Euphausiid larvae. Predictions were not made for April due to gaps in the observed data. (TIF) [file pone.0249818.s007.tif]

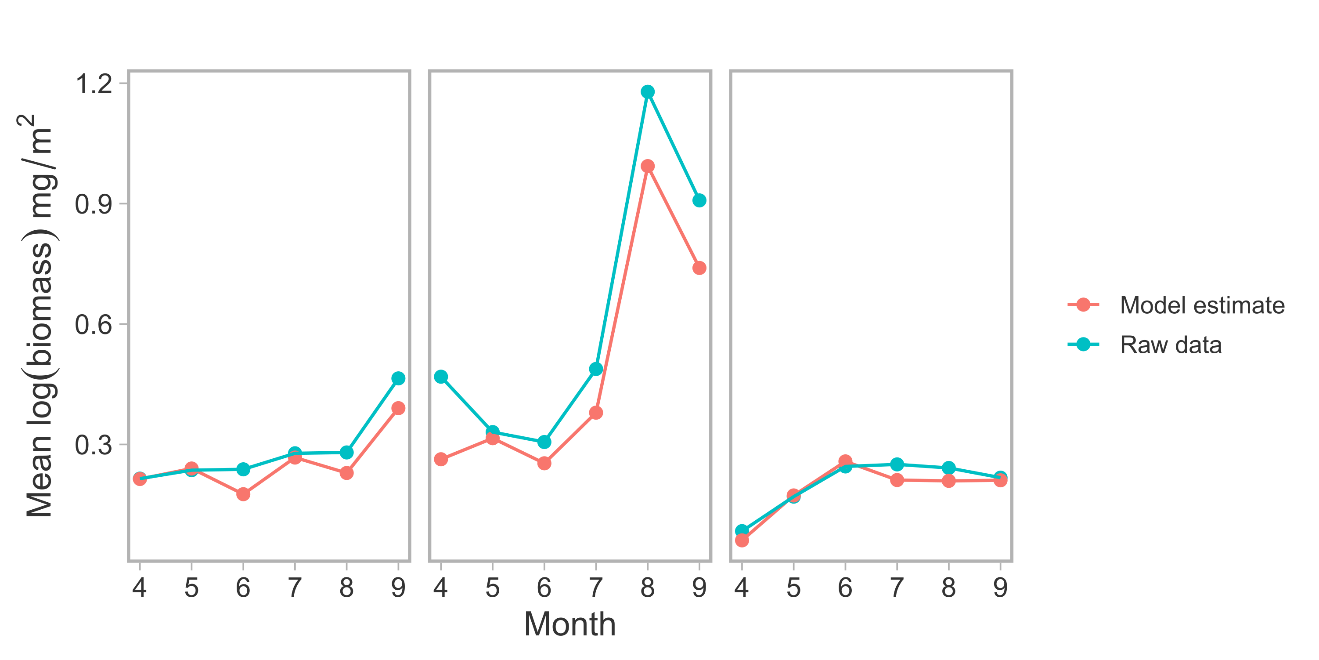

Supplement: S7 Fig — Seasonal comparison in mean observed vs. mean model estimates of biomass for each of the three models for E. pacifica, T. spinifera, and euphausiid larvae (left to right). (TIF) [file pone.0249818.s008.tif]
